# IEEE P7001: A Proposed Standard on Transparency

**DOI:** 10.3389/frobt.2021.665729

**Published:** 2021-07-26

**Authors:** Alan F. T. Winfield, Serena Booth, Louise A. Dennis, Takashi Egawa, Helen Hastie, Naomi Jacobs, Roderick I. Muttram, Joanna I. Olszewska, Fahimeh Rajabiyazdi, Andreas Theodorou, Mark A. Underwood, Robert H. Wortham, Eleanor Watson

**Affiliations:** ^1^ Bristol Robotics Laboratory, UWE Bristol, Bristol, United Kingdom; ^2^ Computer Science and AI Laboratory (CSAIL), MIT, Cambridge, MA, United States; ^3^ Department of Computer Science, University of Manchester, Manchester, United Kingdom; ^4^ NEC Corporation, Tokyo, Japan; ^5^ Department of Computer Science, Heriot-Watt University, Edinburgh, United Kingdom; ^6^ ImaginationLancaster, Lancaster Institute for Contemporary Arts, University of Lancaster, Lancaster, United Kingdom; ^7^ Fourth Insight Ltd, Ewhurst, United Kingdom; ^8^ School of Computing and Engineering, University of the West of Scotland, Paisley, United Kingdom; ^9^ Department of Mechanical and Industrial Engineering, University of Toronto, Toronto, ON, Canada; ^10^ Department of Computing Science, Umeå University, Umeå, Sweden; ^11^ Synchrony Financial, Stamford, CT, United States; ^12^ Department of Electronic and Electrical Engineering, University of Bath, Bath, United Kingdom; ^13^ Nell Watson Ltd., Carrickfergus, United Kingdom

**Keywords:** transparency, explainability, autonomous systems, robot ethics, AI ethics

## Abstract

This paper describes IEEE P7001, a new draft standard on transparency of autonomous systems^1^. In the paper, we outline the development and structure of the draft standard. We present the rationale for transparency as a measurable, testable property. We outline five stakeholder groups: users, the general public and bystanders, safety certification agencies, incident/accident investigators and lawyers/expert witnesses, and explain the thinking behind the normative definitions of “levels” of transparency for each stakeholder group in P7001. The paper illustrates the application of P7001 through worked examples of both specification and assessment of fictional autonomous systems.

## 1 Introduction

There is broad agreement in the AI and robot ethics community about the need for autonomous and intelligent systems to be transparent; a survey of ethical guidelines in AI (Jobin et al., 2019) reveals that transparency is the most frequently included ethical principle, appearing in 73 of the 84 (87%) sets of guidelines surveyed. It is clear that transparency is important for at least three reasons: 1) autonomous and intelligent systems (AIS) can, and do, go wrong, and transparency is necessary to discover how and why; 2) AIS need to be understandable by users, and 3) without adequate transparency, accountability is impossible.

It is important to note that transparency does not come for free. Transparency and explainability are properties that AIS may have more or less of, but these properties are not hardwired–they must be included by design. However, sometimes transparency might be very difficult to design in, for instance in “black box” systems such as those based on Artificial Neural Networks (including Deep Machine Learning systems), or systems that are continually learning.

This paper describes IEEE P7001, a new draft standard on transparency (IEEE, 2020). P7001 is one of the P70XX series of “human standards” emerging from the IEEE Standards Association global initiative on the ethics of autonomous and intelligent systems (IEEE, 2019b). For an overview, see Winfield (2019).

In this paper, we outline the development and structure of P7001. We present the rationale for both transparency and explainability as measurable, testable properties of autonomous systems. We introduce the five stakeholder groups in P7001: users, the general public and bystanders, safety certification agencies, incident/accident investigators and lawyers/expert witnesses. For each of these stakeholders, we outline the structure of the normative definitions of “levels” of transparency.

We will show how P7001 can be applied to either assess the transparency of an existing system–a process of System Transparency Assessment (STA)–or to specify transparency requirements for a system prior to its implementation–a process of System Transparency Specification (STS). We will illustrate the application of P7001 through worked examples of both the specification (STS) and assessment (STA) of fictional autonomous systems.

This paper proceeds as follows. In Section 2, we briefly survey the literature on transparency and explainability as a prelude to, in Section 3, introducing and justifying the definitions for transparency and explainability in P7001. Section 3 also describes the scope and structure of P7001 including each of the five stakeholder groups, and the way P7001 approaches the challenge of setting out testable, measurable levels of transparency for each of these stakeholder groups. In Section 4, we describe how P7001 may be used through the two processes of System Transparency Assessment (STA) and System Transparency Specification (STS), then outline case studies for each, in order to illustrate the application of P7001. Section 5 concludes the paper with a discussion of both the value and the limits of P7001.

## 2 Related Work

The term transparency emerged in the 1990s in the context of information management (Ball, 2009). Nowadays, transparency has become of prime importance in the design and development of autonomous systems (Alonso and de la Puente, 2018), intelligent systems (Olszewska, 2019) as well as human-machine teaming (Tulli et al., 2019; Vorm and Miller, 2020) and human-robot interactions (HRI) (Cantucci and Falcone, 2020).

Transparency can be defined as the extent to which the system discloses the processes or parameters that relate to its functioning (Spagnolli et al., 2016). Transparency can also be considered as the property that makes it possible to discover how and why the system made a particular decision or acted the way it did (Chatila et al., 2017), taking into account its environment (Lakhmani et al., 2016). Indeed, at the moment, there is no single definition of transparency in the literature (Theodorou et al., 2017; Larsson and Heintz, 2020), as it varies depending on its application domain (Weller, 2019) and its dimensions (Bertino et al., 2019). The notion of transparency is also often interwoven with other related concepts such as fairness (Olhede and Rodrigues, 2017), trustworthiness (Wortham, 2020; Nesset et al., 2021), interpretability (Gilpin et al., 2018), accountability (Koene et al., 2019), dependability (TaheriNejad et al., 2020), reliability (Wright et al., 2020), and/or safety (Burton et al., 2020).

The closely related study of explainability has become popular in recent years with the rise of Artificial Intelligence (AI) and AI-based systems (Adadi and Berrada, 2018; Baum et al., 2018; Gunning et al., 2019). This has led to the new field of explainable AI (XAI) (Barredo Arrieta et al., 2020; Confalonieri et al., 2021), which is concerned with the ability to provide explanations about the mechanisms and decisions of AI systems (Doshi-Velez and Kim, 2017; Lipton, 2018).

Current research in XAI focuses on the development of methods and techniques to understand and verify AI-based autonomous and/or intelligent systems (Páez, 2019; Dennis and Fisher, 2020). Explaining AI applications, especially those involving Machine Learning (ML) (Holzinger, 2018), and Deep Neural Networks (DNN) (Angelov and Soares, 2020; Booth et al., 2021), is howbeit still an ongoing effort, due to the high complexity and sophistication of the processes in place (e.g., data handling, algorithm tuning, etc.) as well as the wide range of AI systems such as recommendation systems (Zhang and Chen, 2020), human-agent systems (Rosenfeld and Richardson, 2019), planning systems (Chakraborti et al., 2020), multi-agent systems (Alzetta et al., 2020), autonomous systems (Langley et al., 2017), or robotic systems (Anjomshoae et al., 2019; Rotsidis et al., 2019).

## 3 P7001 Scope and Structure

The aim of P7001 is to provide a standard that sets out “measurable, testable levels of transparency, so that autonomous systems can be objectively assessed and levels of compliance determined” (IEEE, 2020). An autonomous system is defined in P7001 as “a system that has the capacity to make decisions itself, in response to some input data or stimulus, with a varying degree of human intervention depending on the system’s level of autonomy”.

The intended users of P7001 are specifiers, designers, manufacturers, operators and maintainers of autonomous systems. Furthermore P7001 is generic; it is intended to apply to all autonomous systems including robots (autonomous vehicles, assisted living robots, drones, robot toys, etc.), as well as software-only AI systems, such as medical diagnosis AIs, chatbots, loan recommendation systems, facial recognition systems, etc. It follows that P7001 is written as an “umbrella” standard, with definitions of transparency that are generic and thus applicable to a wide range of applications regardless of whether they are based on algorithmic control approaches or machine learning.

### 3.1 Defining Transparency in P7001

The UK’s Engineering and Physical Science Research Council (EPSRC) Principles of Robotics–the first national-level policy on AI–states, as principle four: “Robots are manufactured artifacts. They should not be designed in a deceptive way to exploit vulnerable users; instead their machine nature should be transparent” (Boden et al., 2017). The EPSRC definition of transparency emphasises, through contrast, that transparency in robotics means that the end user is well aware of the manufactured and thus artificial nature of the robot.

Since the release of the EPSRC Principles of Robotics, numerous guidelines and other soft policy declarations have been released by governmental, intergovernmental, non-governmental, and private organisations where transparency is one of the most mentioned ethical principles (Jobin et al., 2019). Yet, each provides its own–vague–definition. For example, the European Commission’s High-Level Expert Group (HLEG) on their “Guidelines for Trustworthy AI” considers transparency to be one of its seven key principles and defines it as a combination of three elements: traceability, explainability, and communication (EC, 2018). Another prominent intergovernmental organisation, the OECD, in its AI ethics guidelines considers transparency as the means of understanding and challenging the outcomes of decisions made by intelligent systems (OECD, 2019). As we saw in the previous section, a similar disagreement exists in the academic literature, where each scholar in transparency-related research has their own definition.

Arguably there can be no universally accepted definition for any given ethical value (Theodorou and Dignum, 2020). Instead, as P7001 is self-contained, an actionable and explicit definition of transparency is required. Thus P7001 defines transparency as “the transfer of information from an autonomous system or its designers to a stakeholder, which is honest, contains information relevant to the causes of some action, decision or behavior and is presented at a level of abstraction and in a form meaningful to the stakeholder.”

P7001 recognises that AI technology cannot be separated from the larger socio-technical system of which it is a component, hence the explicit reference to the designers of the system as responsible agents in providing relevant information. That information, depending on the stakeholder to whom it is targeted, can be anything from records of development decisions to interactive manuals. Further, the keyword *honest* emphasises that only information that is neither false or deceptive can be considered as compliant to the standard.

Furthermore “to consider an autonomous system transparent to inspection, the stakeholder should have the ability to request meaningful explanations of the system’s status either at a specific moment or over a specific period or of the general principles by which decisions are made (as appropriate to the stakeholder)” (Theodorou et al., 2017). This allows the consideration of transparency not only as a *real-time* property, but also as the means of ensuring *traceability* for past events to aid incident investigators (Winfield et al., 2021) and when necessary ensure accountability (Bryson and Theodorou, 2019).

As with transparency, there are multiple definitions for explainability in the literature (Barredo Arrieta et al., 2020). P7001 defines explainability as “the extent to which the internal state and decision-making processes of an autonomous system are accessible to non-expert stakeholders”. Again, this is not an attempt to provide a universally-accepted definition, but rather a *workable* one. The relationship between transparency and explainability in P7001 is that the latter is transparency that is *accessible* to non-experts. In P7001 explainability is a subset of transparency. P7001 defines explainability to stay close to existing literature, while also taking into consideration the multi-stakeholder approach and the wide spectrum of autonomous systems the standard is meant to cover. Thus its normative requirements aim to satisfy both definitions of transparency and explainability. It is also important to note that providing an explanation does not necessarily make a system’s actions completely transparent (De Graaf and Malle, 2017).

### 3.2 Transparency Is Not the Same for Everyone

Transparency is not a singular property of systems that would meet the needs of all stakeholders. In this regard, transparency is like any other ethical or socio-legal value (Theodorou et al., 2017). Clearly a naive user does not require the same level of understanding of a robot as the engineer who repairs it. By the same reasoning, a naive user may require explanations for aspects of reasoning and behaviour that would be obvious and transparent to developers and engineers.

P7001 defines five distinct groups of stakeholders, and AIS must be transparent to each group, in different ways and for different reasons. These stakeholders split into two groups: non-expert end users of autonomous systems (and wider society), and experts including safety certification engineers or agencies, accident investigators, and lawyers or expert witnesses. Stakeholders are beneficiaries of the standard, as distinct from users of the standard: designers, developers, builders and operators of autonomous systems.

Let us now look at the transparency needs of each of these five groups.

#### 3.2.1 Transparency for End Users

For users, transparency (or explainability as defined in P7001) is important because it both builds and calibrates confidence in the system, by providing a simple way for the user to understand what the system is doing and why.

Taking a care robot as an example, transparency means the user can begin to predict what the robot might do in different circumstances. A vulnerable person might feel very unsure about robots, so it is important that the robot is helpful, predictable—never does anything that frightens them—and above all safe. It should be easy to learn what the robot does and why, in different circumstances.

An explainer system that allows the user to ask the robot “why did you do that?” (Sheh, 2017; Chiyah Garcia et al., 2018; Winfield, 2018; Koeman et al., 2020) and receive a simple natural language explanation could be very helpful in providing this kind of transparency^2^. A higher level of explainability might be the ability to respond to questions such as “Robot: what would you do if I fell down?” or “Robot: what would you do if I forget to take my medicine?” The robot’s responses would allow the user to build a mental model of how the robot will behave in different situations.

#### 3.2.2 Transparency for the Wider Public and Bystanders

Robots and AIs are disruptive technologies likely to have significant societal impact (EC, 2018; Wortham, 2020). It is very important therefore that the whole of society has a basic level of understanding of how these systems work, so we can confidently share work or public spaces with them. That understanding is also needed to inform public debates—and hence policy—on which robots/AIs are acceptable, which are not, and how they should be regulated.

This kind of transparency needs public engagement, for example through panel debates and science cafés, supported by high quality documentaries targeted at distribution by mass media (e.g., YouTube and TV), which present emerging robotics and AI technologies and how they work in an interesting and understandable way. Balanced science journalism—avoiding hype and sensationalism—is also needed.

For this stakeholder group, P7001 defines levels of transparency starting with a requirement that follows a proposed *Turing Red Flag* law: “An autonomous system should be designed so that it is unlikely to be mistaken for anything besides an autonomous system, and should identify itself at the start of any interaction with another agent.” (Walsh, 2016). Successive levels build upon this by requiring that systems provide warnings and information about data collected or recorded, since data on bystanders may well be captured.

#### 3.2.3 Transparency for Safety Certifiers

For safety certification of an AIS, transparency is important because it exposes the system’s decision making processes for assurance and independent certification.

The type and level of evidence required to satisfy a certification agency or regulator that a system is safe and fit for purpose depends on how critical the system is. An autonomous vehicle autopilot requires a much higher standard of safety certification than, say, a music recommendation AI, since a fault in the latter is unlikely to endanger life. Safe and correct behaviour can be tested by verification, and fitness for purpose tested by validation. Put simply, verification asks “is this system right?” and validation asks “is this the right system?”.

At the lowest level of transparency, certification agencies or regulators need to see evidence (i.e., documentation) showing how the designer or manufacturer of an AIS has verified and validated that system. This includes as a minimum a technical specification for the system. Higher levels of transparency may need access to source code and all materials needed (such as test metrics or benchmarks) to reproduce the verification and validation processes. For learning systems, this includes details of the composition and provenance of training data sets.

#### 3.2.4 Transparency for Incident/Accident Investigators

Robots and other AI systems can and do act in unexpected or undesired ways. When they do it is important that we can find out why. Autonomous vehicles provide us with a topical example of why transparency for accident investigation is so important. Discovering why an accident happened through investigation requires details of the situational events leading up to and during the accident and, ideally, details of the internal decision making process in the robot or AI prior to the accident (Winfield et al., 2021).

Established and trusted processes of air accident investigation provide an excellent model of good practice for AIS–processes, which have without doubt contributed to the outstanding safety record of modern commercial air travel (Macrae, 2014). One example of best practice is the aircraft Flight Data Recorder, or “black box”; a functionality we consider essential in autonomous systems (Winfield and Jirotka, 2017).

#### 3.2.5 Transparency for Lawyers and Expert Witnesses

Following an accident, lawyers or other expert witnesses who have been obliged to give evidence in an inquiry or court case or to determine insurance settlements, require transparency to inform their evidence. Both need to draw upon information available to the other stakeholder groups: safety certification agencies, accident investigators and users. They especially need to be able to interpret the findings of accident investigations.

In addition, lawyers and expert witnesses may well draw upon additional information relating to the general quality management processes of the company that designed and/or manufactured the robot or AI system. Does that company, for instance, have ISO 9001 certification for its quality management systems? A higher level of transparency might require that a designer or manufacturer provides evidence that it has undertaken an ethical risk assessment of a robot or AI system using, for instance, BS 8611 *Guide to the ethical design of robots and robotic systems* (BSI, 2016).

### 3.3 Measurable and Testable Transparency

Standards generally belong to one of two categories: those that offer guidelines or those that set out requirements. P7001 falls into the latter category. P7001 describes a set of normative requirements, which must be met in order for a given system, its documentation, and the processes used to design and test it, to be labeled as “compliant”.

A major challenge in drafting P7001 was how to express transparency as something measurable and testable. At first this might seem impossible given that transparency is not a singular physical property of systems, like energy consumption. However, when one considers that the degree to which an end user can understand how a system operates will depend a great deal on the way that user documentation is presented and accessed; or the extent to which an accident investigator can discover the factors that led up to an accident can vary from impossible (to discover) to a very detailed timeline of events, it becomes clear that transparency can be expressed as a set of testable thresholds.

It was on this basis that early in the development of P7001 a scale of transparency from 0 (no transparency) to 5 (the maximum achievable level of transparency) was decided upon, for each of the five stakeholder groups outlined above. At the heart of P7001 are five sets–one set for each stakeholder group–of normative definitions of transparency, for each of the levels 1 to 5. Each definition is a requirement, expressed as a qualitative property of the system. In each case the test is simply to determine whether the transparency property required by a given level for a given stakeholder group is demonstrably present or it is not. The choice of five levels was determined as a compromise between a reasonable level of granularity while allowing for discernible differences between successive levels.

Having established a general approach to measurable, testable levels of transparency, the P7001 working group then faced the key question “should those discrete transparency levels be written to reflect the transparency properties found in present day autonomous systems, or should they instead go beyond the present state of the art?” Phrased in another way, should P7001 be written such that most well-designed present day autonomous systems achieve a high level of compliance, or instead as a standard that stretches designers beyond current good practice? Taking a cue from the IEEE P70XX series of standards-in-development, which–in expressing human (ethical) concerns as standards for guidance or compliance–go well beyond the scope of traditional standards, it was determined that P7001 should similarly aim to challenge and extend the practice of transparency.

Given increasingly rapid advances in the capabilities of AI systems, it was also felt that P7001 should consider likely near, and to some extent, medium term advances in the state of the art (for instance, explainable AI for machine learning). However, the working group did not take account of possible long term advances, such as artificial general intelligence or machine consciousness. As and when it becomes necessary, the standard can be updated to meet with advances in the state of the art.

The general principle was, therefore, established that transparency levels should start (on level 1) with transparency measures that we might generally expect to find in well-designed present day systems, or that could be easily provided. Levels 2 and up should be successively more demanding, going beyond what one would presently expect in most well designed systems, and in some cases require solutions that are—at the time of writing—the subject of ongoing research.

The approach outlined above is illustrated below in Tables 1, 2 for the stakeholder groups “end users” and “accident investigators”, respectively, (The illustrations in Tables 1, 2 are abbreviated versions of the transparency definitions for end users and accident investigators in P7001 for robots only, rather than autonomous systems in general).

**TABLE 1:** Transparency levels for end users.

Transparency levels (Non-cumulative)	Definition
0	None
1	A user manual must be provided, which sets out how a robot will behave in different circumstances
2	The user manual should be presented as an interactive visualisation or simulation
3	The robot should be equipped with a “why did you just do that?” function which, when activated, provides the user with an explanation of its previous action, either as displayed or spoken text koeman et al. (2020)
4	The robot should be equipped with a “what would you do if … ?” function
5	Not defined

**TABLE 2:** Transparency levels for accident investigators.

Transparency levels (Cumulative)	Definition
0	None
1	The robot should be fitted with a recording device to allow capture and playback of the situation around it, leading up to and during an accident
2	The robot should be equipped with a data logging system capable of recording a date and time stamped record of robot sensor inputs, user commands, and actuator outputs
3	As level 2, except that the data logging system should conform to an existing open or industry standard, and additionally log high level decisions
4	As level 3, except that the data logging system should also log the reasons for the robot’s high level decisions
5	In addition to level 4, the robot’s designers should provide accident investigators with tools to help visualise the robot’s data log

In Tables 1, 2, we see that each level *n* describes a successively greater degree of transparency than the previous level n−1. For most stakeholder groups each level builds upon previous levels, so if a system meets level *n*, then it also meets levels n−1, etc. Thus transparency levels are cumulative for accident investigators in Table 2, but not in Table 1 for end users so, for instance, a designer may choose to provide an interactive visualisation of level 2 instead of the user manual of level 1 (or they may choose to provide both).

Level 1 in Table 1–a user manual–will typically be present for all present day robots. Similarly, the recording device required by level 1 in Table 2 will be easy to provide, if not already present.

Consider now levels 2–4 in Table 1 for end users. Level 2–an interactive visualisation–is more demanding than level 1 but perfectly feasible with current simulation and visualisation technology. Levels 3 and 4 do, however, go beyond the current state of the art in robotics, but methods for implementing this kind of explainability in robots are emerging (Theodorou et al., 2017; Winfield, 2018; Rosenfeld and Richardson, 2019; Koeman et al., 2020; Dennis and Oren, 2021).

Consider also levels 2–5 in Table 2, for accident investigators. Level 2–a bespoke data logging system, while not currently present in many robots, would not be technically challenging to implement. Winfield and Jirotka (2017) provide a general outline of what is required. Level 3–a data logging system conforming to an existing standard is more challenging since, for robots in general, such standards do not yet exist^3^. For autonomous vehicles, however, a closed standard for automotive Event Data Recorders does exist in (SAE J1698_2017), with another in development (IEEE P1616). Level 4 goes further in requiring the data logger to record the reasons for high level decisions; something that would require access to internal processes of the robot’s control system, which would not normally be accessible via, for instance, the robot’s API.

### 3.4 Compliance

A system would be compliant with P7001 if it meets at least level 1 transparency for at least one stakeholder group. Note, however, that a simple statement that “system x is compliant with P7001” would be misleading. The correct way to describe P7001 compliance is through the multi-element description of the STA, outlined in Section 4.

Consider a system that is assessed as providing level 1 transparency for one stakeholder group only: the absolute minimum level of compliance. In some bounded and benign use cases, such a level might still be regarded as adequate. However, what constitutes sufficient or appropriate levels of transparency will vary a great deal from one system and its intended use to another.

It is important also to recognise that stakeholder groups and their transparency requirements are independent of each other, thus there is no expectation that if a system meets a particular level in one stakeholder group, it should also meet the same level in other groups.

In practice, the decision over which transparency level is needed in each stakeholder group should be guided by an ethical risk assessment. BS 8611 sets out a method for ethical risk assessment of robots or robotic systems (BSI, 2016), and an example of ethical risk assessment for a child’s toy robot can be found in Winfield and Winkle (2020). Example scenarios will be outlined in Section 4 below.

It is clear that 1) compliance with P7001 will vary a great deal between systems, and between stakeholder groups for a particular system, and 2) whether the level of compliance for a given system is adequate or not will depend on the possible risk of (ethical) harms should the system fail or be compromised. So we might expect that, in general, safety-critical autonomous systems would require higher levels of transparency than non-critical systems. One thing we can be reasonably sure of is that a system that fails to score even level 1 for any stakeholder group is unlikely to have adequate transparency.

## 4 P7001 Processes

P7001 is a process standard; it does not specify *how* the transparency measures defined in it must be implemented, only the kind of transparency each measure affords and how to determine whether it is present or not. Some transparency measures will require designers to include well understood features; transparency for accident investigators, for instance, requires that systems incorporate event data recorders (EDRs)–the functional equivalent of aircraft flight data recorders–without which it would be impossible to investigate accidents. The draft standard does not, however, specify required functionality of the EDR, except at a very generic level.

As mentioned above, P7001 has two primary functions. The first is as a tool for assessing the transparency of existing systems, called a System Transparency Assessment (STA), and the second as a guide for creating a transparency specification for a given system prior to, or during, its design: this is a System Transparency Specification (STS). Each of these will now be illustrated with a case study.

### 4.1 System Transparency Assessment for a Robot Toy

In Winfield and Winkle (2020), we describe an ethical risk assessment for a fictional intelligent robot teddy bear we called RoboTED. Let us now assess the transparency of the same robot. In summary, RoboTED is an Internet (WiFi) connected device with cloud-based speech recognition and conversational AI (chatbot) with local speech synthesis; RoboTED’s eyes are functional cameras allowing the robot to recognise faces; RoboTED has touch sensors, and motorised arms and legs to provide it with limited baby-like movement and locomotion—not walking but shuffling and crawling.

Our ethical risk assessment (ERA) exposed two physical (safety) hazards including tripping over the robot and batteries overheating. Psychological hazards include addiction to the robot by the child, deception (the child coming to believe the robot cares for them), over-trusting of the robot by the child, and over-trusting of the robot by the child’s parents. Privacy and security hazards include weak security (allowing hackers to gain access to the robot), weak privacy of personal data especially images and voice clips, and no event data logging making any investigation of accidents all but impossible^4^.

The ERA leads to a number of recommendations for design changes. One of those is particularly relevant to the present paper: the inclusion of an event data recorder, so our outline transparency assessment, given below in Table 3, will assume this change has been made.

**TABLE 3:** Outline system transparency assessment (STA) for RoboTED.

Stakeholder Group	Transparency level(s)	Evidenced by
[i] users	1, 2	A user manual is provided for parents. As well as detailing how parents can show children how best to use RoboTED, the manual explains the risks (addiction, deception and over-trusting) and how to minimise these. The manual also shows how to guard against hacking and check personal data has been deleted (level 1). An interactive online visual guide is also provided, for both parents and children (level 2)
[ii] general public	1	P7001 level 1 requires that a robot identifies itself as an autonomous system, following Walsh (2016). When powered up, or on waking from sleep mode, RoboTED announces itself as a robot
[iii] certification agencies	2	RoboTED has been certified as safe against standard EU EN 62115 (2020) *Safety of Electric Toys*, and descriptions of the system and how it has been validated are available for safety certifiers. This meets P7001 level 2
[iv] accident Investigators	2	The robot is equipped with a data logging system as outlined in Table 2
[v] lawyers and expert witnesses	2	P7001 level 2 requires that a system has been subjected to an ethical risk assessment, which can be made available to lawyers or expert witnesses. This is the case for RoboTED

### 4.2 System Transparency Specification for a Vacuum Cleaner Robot

Consider now a fictional company that designs and manufactures robot vacuum cleaners for domestic use. Let us call this company nextVac. Let us assume that nextVac is well established in the domestic market and has a reputation both for the quality of its products and responsible approach to design and manufacture. nextVac now wishes to develop a new line of robot vacuum cleaners for use in healthcare settings: including hospitals, clinics and elder care homes.

nextVac begins the design process with a scoping study in which they visit healthcare facilities and discuss cleaning needs with healthcare staff, facilities managers and cleaning contractors. Mindful of the additional safety, operational and regulatory requirements of the healthcare sector (over and above their domestic market), nextVac decides to capture the transparency needs of the new product–while also reflecting the findings of the scoping study–in a System Transparency Specification (STS), guided by IEEE P7001. Their intention is to follow the STS with an initial product design specification. In turn this specification will be subjected to an Ethical Risk Assessment (ERA), guided by BS8611. Depending on the findings of the ERA, the company will iterate this process until a product specification emerges that is technically feasible, tailored to customer needs, and addresses both ethical risks and transparency needs.

Capturing the full process of drafting an STS for this scenario is beyond the scope of this paper, so instead we outline the key requirement in Table 4.

**TABLE 4:** Outline system transparency specification (STS) for nextVac.

Stakeholder Group	Transparency level(s) Required	Rationale
[i] users	1, 2 (see Table 1)	A comprehensive user manual is required, covering both use and maintenance. The manual should be written in compliance with standard IEC/IEEE std 82,079 *Preparation of information for use*, as recommended by P7001 (level 1). An interactive online visual guide is also required, for both operators of the cleaning robot and facilities managers (level 2). Levels 3 and 4 are not required as the robot is not expected to need a complex human robot interface. The robot will only require a limited number of behaviours and these will be indicated by warning lights and sounds, see group [ii] below
[ii] general public	1, 2	The robot’s design will ensure that its machine nature is apparent; lights and sounds will provide simple audio-visual indications of what the robot is doing at any time (level 1). The robot will provide physical cues showing the location of sensors, and publicly available information will explain what data is stored and why (see [iv] accident Investigators in this table), and that this data will not include any personal data (level 2)
[iii] certification agencies	3	The robot will be certified as safe against relevant standards, such as ISO 10218 (2011) (noting that ISO 10218 is a generic standard for the safety of industrial robots). Descriptions of the system and how it has been validated will be made available to safety certifiers (level 2). In addition, a high level model (simulation) of the robot will be developed and made available (level 3)
[iv] accident Investigators	3 (see Table 2)	The robot will be equipped with a data logging system, which records high level decisions (as outlined in Table 2). Noting that the data logging system will not record any personal data. Levels 4 and 5 are not considered essential, as the cleaning robot will only require a limited number of behaviours, nor will it learn
[v] lawyers and expert witnesses	4	nextVac already has certification of quality management (QM) to standard ISO 9001 (level 1). Ethical risk assessment (ERA) against BS8611 will be undertaken (level 2). nextVac has in place processes of ethical governance (level 3). nextVac also maintains complete audit trails for QM, ERA and ethical governance processes (level 4)

The outline STS for nextVac’s proposed new vacuum cleaning robot for healthcare, leads to a number of clear technical design requirements, especially for stakeholder groups [i], [ii], and [iv], alongside process requirements for groups [iii] and [v]. The STS will thus feed into and form part of the product design specification.

Note also that the outline STS in Table 4 illustrates–for groups [i] and [iv]–the value of also asking the question, and therefore seeking explicit justification, for why certain higher levels of transparency are *not* required.

## 5 Concluding Discussion

In this concluding section, we first discuss security, privacy and transparency before then outlining and discussing the challenges faced when drafting P7001, and its limitations.

### 5.1 Security, Privacy and Transparency

Security and privacy practices are generally embedded within the fabric of autonomous systems. Security standards, especially for regulated industries such as transportation, utilities and finance, receive particular attention by system architects and auditors, but transparency within these mature frameworks tends to be addressed indirectly. To adequately consider transparency for security and privacy, STA and STS statements must be tied closely to prevailing information security standards.

The STA equivalent in security standards such as ISO 27001 and NIST 800-53 tends to be framed as governance or assurance tasks (NIST, 2020). These tasks, both automated and manual, verify the presence of a security control. For instance, in P7001 example scenario B.6 (Medical Decision Support), an assurance task verifies that patient information is encrypted in transit and in rest and is not exposed beyond a circumscribed list of providers. An autonomous system whose security and privacy protections are transparent will disclose the methods being used to protect sensitive information. In some cases, users can perform assurance tasks themselves.

Autonomous system architects can fashion STAs following recommendations of the NIST Big Data Reference Architecture (SP 1500-r2) wherein higher security and privacy safety levels provide additional disclosures–i.e., transparency–via multiple techniques including a System Communicator. NIST 1500-r2 addresses three voluntary levels of system transparency, each of which can be integrated into an STS (Chang et al., 2019, Sect. 2.4.8). Big data plays an increasingly prominent role in autonomous systems and presents particularly challenging security and privacy risks.

Newer autonomous systems constructed using DevOps principles offer additional opportunities to embed STS requirements. IEEE 2675–2021 cites benefits for DevOps communities: “Transparency prioritizes ease of visibility, availability, reachability, and accessibility of information and actions between entities, people, or systems” (IEEE, 2021, Sect. 5.3.3).

Some facets of security and privacy are global and human, affecting well-being in ways that require different and novel metrics. IEEE 7010–2019 directly cites the relevance of P7001 and further recommends that autonomous data collection plans address “…issues related to collection and use of data, such as ethics, *transparency*, data privacy, data governance, security, protection of data, nudging, coercion, algorithmic bias, asymmetry, and redundancy … ” (IEEE, 2019a, Sect. 5.3.1, Table 6, italics added).

### 5.2 Challenges and Limitations

P7001 is, to the best of our knowledge, the first attempt to write a standard on transparency; this alone would make development of the standard challenging. In particular:

(1) The comparative youth of the field makes it difficult to assess what it is practical to require now in terms of transparency, let alone what might be practical within the lifetime of the standard. This is acute in the case of Deep Neural Nets (DNNs), which many people wish to use but also present a challenge to explainability (at least), if not necessarily to transparency in general.
(2) The heterogeneous nature of transparency is a problem. Is the simple provision of information (e.g., a log) sufficient, or must the information be in a contextualised form (e.g., an explanation)? Across and within the stakeholder groups, there was discussion over whether contextualisation was desirable since it necessarily creates a system-generated interpretation of what is happening, which could introduce biases or errors in reporting. Is something transparent if we can inspect all parts of it but not understand the emergent behaviour, as may be the case for a DNN?
(3) What is the best medium for the presentation of such information? There is a tendency to assume it should be written or verbal but diagrams and other visual mechanisms can also be important. A range of possible outputs increases accessibility, and some outputs may be better suited to certain situations, for example, where privacy is a factor, or an incident where all people nearby must be immediately notified through an alarm.
(4) Within P7001’s various stakeholder groups, it was sometimes difficult to foresee what transparency might be wanted for, and without knowing the purpose of transparency it was hard to determine what should be required and how compliance might be measured.
(5) When might transparency lead to over-confidence? In a recent paper, Kaur et al. (2020) showed that the provision of explainability mechanisms led to over-confidence in a model. This may also contribute to automation bias, a tendency to place unwarranted trust in the accuracy and infallibility of automated systems.
(6) Transparency exists in tension with a number of other ethical principles, most notably security (where lack of transparency is often a first line of defence) and privacy (for instance, in our RoboTED example, some potential explanations might reveal personal information about the child who owned the toy). This highlights the need for determinations about appropriate levels of transparency to be informed by both ethical risk assessment and the practices outlined in Section 5.1.

The challenges mentioned above were further compounded by the demands of writing normative definitions of transparency that are at the same time sufficiently generic to apply to all autonomous systems, while also specific enough to be implemented and expressed with enough precision to allow the question “is this transparency measure present in this system or not” to be answered. P7001 has been drafted as an “umbrella” standard, and an indicator of its success would not only be its application to real world autonomous systems, including both robots and AIs, but also the subsequent development of domain specific variants. Each branching standard, 7001.1, 7001.2, etc., would inherit the generic definitions of 7001 but elaborate these more precisely as, for instance, standards on transparency in autonomous vehicles, transparency of AIS in healthcare, and so on.

To what extent did the difficulties articulated here lead to limitations in P7001? One clear limitation is that P7001 does not offer detailed advice on how to implement the various kinds of transparency described in it. However, we would argue that a strength of P7001 is the clear articulation of the two processes of systems transparency assessment (STA) and specification (STS). Another related limitation is that several definitions of higher levels of transparency require techniques that have not yet been developed–to the extent that they can be readily applied. One example is the requirement for systems to provide non-expert users with answers to “why” and “what if” questions, in levels 3 and 4 of transparency for users. Another example would be higher levels of verification and validation for systems that learn, within the stakeholder group of certification agencies, given that verification of autonomous systems is challenging–especially for machine learning systems–and remains the subject of current research.

These limitations may suggest that there would be no value in assessment of the transparency of autonomous systems that can learn (either offline or online). However, we would argue that there is value, even–and especially–if assessment exposes transparency gaps in machine learning systems. Just as transparency is vitally important, so is honest appraisal of the levels of transparency of a given system. P7001 will, for the first time, allow us to be rigorously transparent about transparency.

## Data Availability

The original contributions presented in the study are included in the article/Supplementary Material, further inquiries can be directed to the corresponding author.

## Footnotes

2 Noting that Winograd’s SHRDLU Natural Language Processing system demonstrated this capability in 1972 (Winograd, 1972)

3 Although at least one open standard for robots is known to be in draft.

4 The ERA also considers environmental risks, including sustainability, repairability and recyclability, but these have no bearing on transparency and do not need to be considered here.
